# HtrA2 Independently Predicts Poor Prognosis and Correlates with Immune Cell Infiltration in Hepatocellular Carcinoma

**DOI:** 10.1155/2023/4067418

**Published:** 2023-01-17

**Authors:** Lei Feng, Zhen Li, Yao Xiong, Ting Yan, Changmin Fu, Qiuyue Zeng, Huamin Wang

**Affiliations:** ^1^The Division of Gastroenterology and Hepatology, Suining Central Hospital, Suining, Sichuan, China; ^2^North Sichuan Medical College, Nanchong, Sichuan, China; ^3^Sichuan Vocational and Technical College, Suining, Sichuan, China; ^4^Zunyi Medical University, Zunyi, Guizhou, China

## Abstract

High-temperature requirement protein A2 (HtrA2), a mitochondrial protein, is related to apoptosis regulation. However, the role of HtrA2 in hepatocellular carcinoma (HCC) remains unclear. In the present study, we explored the prognostic value and expression pattern of HtrA2 in HCC and confirmed its independent value for predicting outcomes via Cox analyses. LinkedOmics and GEPIA2 were used to construct the coexpression and functional networks of HtrA2. Additionally, the data obtained from TCGA was analyzed to investigate the relationship between the infiltration of immune cells and HtrA2 mRNA expression. Finally, the expression pattern of HtrA2 in HCC was confirmed by wet-lab experiments. The results showed high HtrA2 expression (*P* < 0.001) presented in tumor tissues in TCGA-HCC. Moreover, high HtrA2 expression was confirmed to be associated with poor HCC patient survival (*P* < 0.05). HtrA2 has also been recognized as an essential risk factor for overall survival (*P*=0.01, HR = 1.654, 95% CI 1.128–2.425), disease-specific survival (*P*=0.004, HR = 2.204, 95% CI 1.294–3.753), and progression-free interval (*P*=0.007, HR = 1.637, 95% CI 1.145–2.341) of HCC. HCC patients with low HtrA2 methylation had worse overall survival than patients with high methylation (*P*=0.0019). Functional network analysis suggests that HtrA2 regulates mitochondrial homeostasis through pathways involving multiple microRNAs and transcription factors in HCC. In addition, HtrA2 expression correlated with infiltrating levels of multiple immune cell populations. At last, increased expression of HtrA2 in HCC was confirmed using wet-lab experiments. Our study provides evidence that the upregulation of HtrA2 in HCC is an independent predictor of prognosis. Our results provide the foundation for further study on the roles of HtrA2 in HCC tumorigenesis.

## 1. Introduction

Liver cancer represents the second leading cause of cancer-related deaths, with one out of every ten cancer-related deaths globally resulting from liver cancer [1]. Hepatocellular carcinoma (HCC) is the most frequent histological type of liver cancer (90% of all liver cancers), and its prognosis is poor [2, 3]. The survival rate of HCC is primarily determined by the stage of diagnosis [4]. Usually, the reported 5-year overall survival rate is only about 18% [5]. When HCC is diagnosed in an early phase, complete tumor resection and liver transplantation have led to improved 5-year survival rates of 47.9% and 59.3%, respectively [6]. Sadly, only a minority of patients with HCC received an early diagnosis. At the same time, a large proportion was diagnosed at an advanced stage, presenting symptoms of local tumor progression and distant metastasis [7–9].

Despite being the primary modality for treating tumor progression and distant metastasis, chemotherapy has shown limited antitumor efficacy in treating HCC due to poor targeting and low drug concentrations in tumor tissue [10–13]. Whereas, due to severe side effects and drug resistance, existing targeted drugs cannot provide satisfactory efficacy in treating HCC [14]. Targeting anti-HCC drugs with precise pharmacokinetics and reliable efficacy is an urgent need. In HCC, relatively little is known regarding the impact of host and genomic factors on the progression of preinvasive lesions [15]. Investigating these effects can improve our understanding of the molecular biology of HCC and favor the development of better screening strategies, improving patient prognosis. However, early diagnosis and targeted therapies for HCC are complex and challenging due to the lack of specific markers associated with the stage or type.

High-temperature requirement protein A2 (HtrA2), a member of the high-temperature requirement family, is a serine protease localized to mitochondria [16]. HtrA2 was originally considered a heat shock-induced serine protease in *Escherichia coli* but is now identified as a mitochondrial proapoptotic protein and participates in the maintenance of mitochondrial homeostasis [17–19]. Multiple reports show that HtrA2 is involved in the development of various cancers, including colorectal, breast, ovarian, and prostate cancer [20–23]. Recent studies have shown that plasma HtrA2 can be used as a clinical diagnostic marker for gastric carcinoma [24]. In addition, the occurrence of hepatocellular carcinoma cell apoptosis is highly dependent on HtrA2 expression [25]. Besides, HtrA2 has also been reported to participate in the suppression of hepatocellular carcinoma cell growth by staurosporine [26]. Previous studies on the relationship between HtrA2 and HCC were too specific and needed a comprehensive view. Moreover, prior studies cannot provide a clear answer regarding whether HtrA2 serves as a predictive biomarker for HCC.

In the present study, we explored the expression, methylation level, and prognostic significance of HtrA2 in HCC patients in The Cancer Genome Atlas (TCGA) and verified our conclusions in multiple independent clinical cohorts. Furthermore, we used a multidimensional analysis to identify the functional enrichment of HtrA2-related genes in HCC and studied the relationship between HtrA2 and tumor immunity. The pertinent findings of this study may serve as the basis for developing new diagnostic and therapeutic strategies to treat HCC.

## 2. Materials and Methods

### 2.1. Data Acquisition

HCC patients' gene expression data, along with their clinical profiles such as gender, age, TNM classification, tumor stage, and survival status, were downloaded from the TCGA database (https://portal.gdc.cancer.gov/). HCC and normal tissue gene expression profiles of GSE121248, GSE101685, GSE76427, and GSE76297 were obtained from the Gene Expression Omnibus (GEO) database in the National Center for Biotechnology Information (NCBI, https://www.ncbi.nlm.nih.gov/geo/).

### 2.2. HtrA2 Differential Expression

In the TCGA-HCC cohort, the receiver operating characteristic (ROC) curve was generated to evaluate the diagnostic accuracy of HtrA2 for HCC, and differential expression analysis of mRNA levels of HtrA2 was performed by the Wilcoxon test, including unpaired and paired tests. The study used R to analyze the differential expression of HtrA2 in the GEO datasets (GSE121248, GSE101685, GSE76427, and GSE76297). We input HtrA2 in the “Gene_DE” module of TIMER2 (Tumor Immune Estimation Resource, Version 2 Web (https://timer.comp-genomics.org/) and evaluated the expression difference of HtrA2 between tumor and normal tissues for the different tumors or specific tumor subtypes of the TCGA project. The associations of HtrA2 expression levels with clinical characteristics were examined using the nonparametric Wilcoxon rank sum test or Kruskal–Wallis test. R software was used for data visualization.

### 2.3. Survival Analyses of HtrA2 and Filtered Immune Cells

Survival analysis in the TCGA-HCC cohorts was performed between high and low HtrA2 expression groups using the Survival package for R, and Kaplan–Meier survival curves were plotted using the survminer package. Kaplan–Meier Plotter (https://kmplot.com/) is a meta-analysis-based tumor biomarker assessment website. The relation between HtrA2 expression and survival in HCC was also analyzed in Kaplan–Meier Plotter. Survival analyses of the filtered immune cells in the TCGA-HCC cohorts were performed by the Kaplan–Meier survival analysis, with a cut-off level set at the median value.

### 2.4. The Independent Predictive Value of HtrA2

The Cox regression analyses were performed to confirm the independent predictive value of HtrA2 in HCC and explore the relationships between important clinical features and prognosis. First, 13 different variables were included in the univariate analysis, and their correlations with prognosis were checked. Then, factors with *P* < 0.1 in univariate analyses were selected for multivariate analyses to evaluate whether each variable has an independent predictive value in HCC.

### 2.5. HtrA2 Methylation Level and Its Prognosis Analysis

The methylation level and copy number variation (CNV) data of HtrA2 were acquired through the cBioPortal web platform (https://www.cbioportal.org/). Furthermore, the correlation between HtrA2 methylation level and HtrA2 gene expression was analyzed using Pearson's correlation analysis. The HtrA2 expression in different copy number variation groups was compared using the Kruskal–Wallis test. The changes in the promoter methylation level of HtrA2in HCC and normal tissues were analyzed using the UALCAN online tool (https://ualcan.path.uab.edu/). The predictive value of the HtrA2 methylation level in HCC was investigated using the MethSurv online tool (https://biit.cs.ut.ee/methsurv/).

### 2.6. Analyses of LinkedOmics and GEPIA2 Databases

LinkedOmics (https://www.linkedomics.org) is a freely accessible website with multiomics data derived from 32 TCGA cancer types. HtrA2 coexpression analysis was performed using the Pearson test in the “LinkFinder” module of LinkedOmics, and its results were plotted as volcano plots and heatmaps. Analyses of Gene Ontology, Kyoto Encyclopedia of Genes and Genomes (KEGG) pathways, miRNA-target enrichment, and transcription factor-target enrichment were performed using Gene Set Enrichment Analysis (GSEA) in “LinkInterpreter” module of LinkedOmics. The rank criterion was false discovery rate (FDR) < 0.05, and simulations were 1000. Survival heatmaps of top coexpression genes were plotted using the GEPIA2 database (a web tool for analyzing the RNA sequencing expression data, https://gepia2.cancer-pku.cn/#correlation).

### 2.7. Analysis of the Relationship between Immune Infiltration and HtrA2 Expression

The ssGSEA (single-sample Gene Set Enrichment Analysis) was applied to quantify the relative tumor infiltration levels of immune cell types in HCC using the R package “GSVA.” The relationship between the infiltration of immune cells and the HtrA2 mRNA expression was evaluated by using the Spearman correlation.

### 2.8. Hepatocellular Carcinoma Tissue Samples and Cell Culture

Tissue samples were obtained from 12 HCC patients without previous HCC treatment who underwent surgery. These patients were recruited from the Suining Central Hospital between 2014 and 2018. Patient consent and institutional ethics approval were obtained. The normal human liver cell line HL7702 and human HCC cell lines HepG2, SMMC7721, and Huh7 were purchased from the ATCC (Manassas, VA, USA). All cells were cultured at 37°C in a 5% CO_2_ atmosphere with 95% air in Dulbecco's Modified Eagle Medium (DMEM, Lonza, NJ, USA) supplemented with 50 U/ml penicillin/streptomycin, 1% nonessential amino acids, and 10% fetal bovine serum (FBS, Lonza, NJ, USA).

### 2.9. Immunohistochemistry (IHC)

All tissue samples were paraffin-embedded and sectioned. Paraffin sections were dewaxed and rehydrated, followed by heat-induced antigen retrieval in boiling sodium citrate (pH = 6). Immediately after blocking, the samples were incubated with rabbit polyclonal anti-HtrA2 (1 : 100, ZEN BIO, China) at 4°C overnight. The immunohistochemistry kit (ZSGB-BIO, China) was used for immunohistochemical staining. Two pathologists reviewed the histologic diagnoses.

### 2.10. Total RNA Isolation and Real-Time PCR

Total RNA was extracted from cell lines using an RNAiso Kit (Takara, Dalian, China) based on the manufacturer's protocol. Next, reverse transcription was carried out using a cDNA synthesis kit (Takara, Dalian, China). Real-time PCR analysis was carried out with SYBR Green in a real-time PCR machine. GAPDH served as an internal reference. The specific primers used were HtrA2 sense: 5′-GGGCAGTGCTGTTGTTGTT-3′ and antisense: 5′-GCAGGTGCTGTCTTCTCCA-3′, GAPDH sense: 5′-GACCCCTTCATTGACCTCAAC-3′ and antisense: 5′-CTTCTCCATGGTGGTGAAGA-3′.

### 2.11. Western Blot Analysis

Total protein from cell lines was extracted using a whole-cell extraction buffer. Protein samples were separated by SDS-PAGE and transferred to polyvinylidene fluoride membranes (Millipore Corporation, Bedford, MA, USA). After blocking, the membranes were incubated overnight at 4°C with the following primary antibodies: HtrA2 (1 : 1000) and GAPDH (1 : 2500). Primary antibody against HtrA2 was purchased from ZEN BIO, China. The primary antibody against GAPDH was purchased from Abcam, Cambridge, UK. Subsequently, the membranes were developed with secondary peroxidase-coupled anti-rabbit antibodies, followed by chemiluminescence imaging. Band intensity was quantified using ImageJ software.

### 2.12. Statistical Analysis

All experiments were repeated at least three times. Rank-based nonparametric Kruskal–Wallis test was used for non-normally distributed data. Statistical analysis of experimental data was performed using SPSS statistical software version 26.0. The statistical significance was considered for *P* < 0.05.

## 3. Results

### 3.1. High HtrA2 Expression in HCC

**Figure 1:**
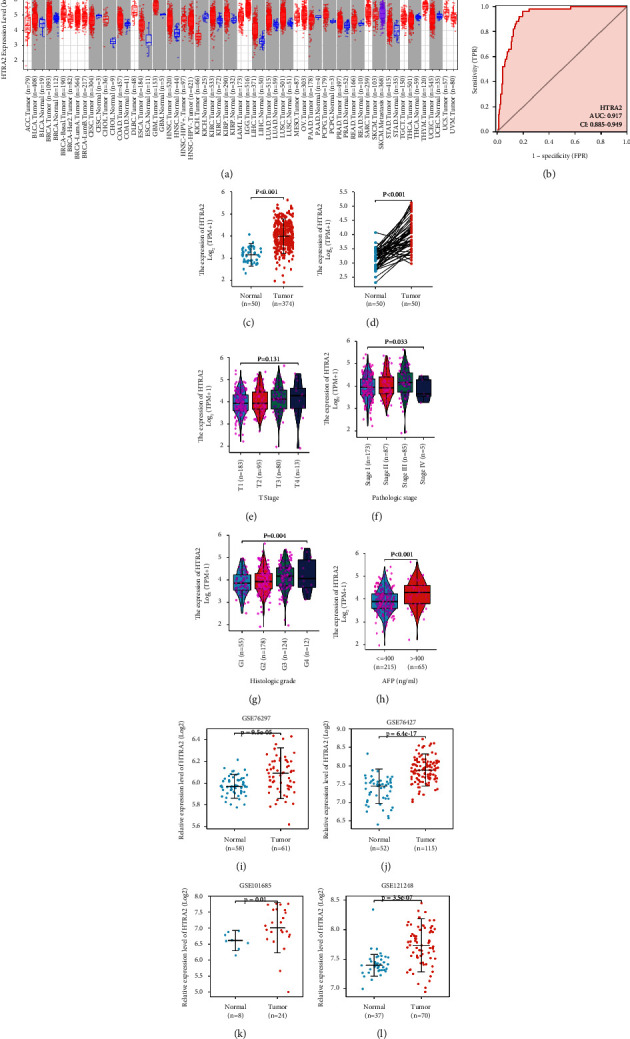
HtrA2 mRNA expression in HCC and other human tumor types from the TCGA and GEO databases. (a) The expression levels of HtrA2 in different types of human tumor tissues from the TCGA database. (b) Receiver operating characteristic analysis (ROC) of HtrA2 in HCC (*n* = 424). (c) Expression levels of HtrA2 in HCC (*n* = 374) and normal tissue (*n* = 50). (d) The HtrA2 expression levels in paired (patient-matched) HCC tumors (*n* = 50) and normal adjacent tissues (*n* = 50). (e) The association of HtrA2 expression and T classification in HCC (*n* = 371). (f) The association of HtrA2 expression and pathologic stage in HCC (*n* = 350). (g) The association of HtrA2 expression and histologic stage in HCC (*n* = 369). (h) The association of HtrA2 expression in HCC and serum alpha-fetoprotein levels (*n* = 280). (i–l) The comparisons of HtrA2 expression levels between normal liver tissues and HCC tissues obtained from the GEO database. HCC: hepatocellular carcinoma; TCGA: the cancer genome atlas; GEO: gene expression omnibus.

Using the TCGA sequencing data, an analysis of HtrA2 mRNA expression in multiple human tumor tissues was performed. The results showed that HtrA2 mRNA expression was significantly increased in various human tumor tissue in the TCGA database compared with that in the normal tissue (Figure 1(a)), including invasive breast carcinoma (BRCA), urothelial bladder carcinoma (BLCA), cholangiocarcinoma (CHOL), colon adenocarcinoma (COAD), esophageal carcinoma (ESCA), glioblastoma multiforme (GBM), head-neck squamous cell carcinoma (HNSC), kidney renal clear cell carcinoma (KIRC), kidney renal papillary cell carcinoma (KIRP), hepatocellular carcinoma (LIHC), lung adenocarcinoma (LUAD), lung squamous cell carcinoma (LUSC), pheochromocytoma and paraganglioma (PCPG), prostate adenocarcinoma (PRAD), rectum adenocarcinoma (READ), stomach adenocarcinoma (STAD), thyroid carcinoma (THCA), and uterine corpus endometrial carcinoma (UCEC).

ROC curve analysis of HtrA2 mRNA expression for the prediction of HCC revealed an area under the ROC curve (AUC) of 0.917 (95% CI: 0.885–0.949) (Figure 1(b)). In the TCGA-HCC cohort, the mRNA expression of HtrA2 in tumor tissue was compared with that in normal or paired adjacent tissues. The paired and unpaired test results indicated that HtrA2 mRNA expression levels in HCC were elevated (all *P* < 0.001) (Figures 1(c) and 1(d)). In HCC, HtrA2 expression significantly correlated with the pathological stage (according to Edmondson–Steiner grade, Figure 1(f)) and histological grade (Figure 1(g)) but not with the T stage (Figure 1(e)). HCC patients with an AFP level higher than 400 ng/mL had significantly higher HtrA2 expression levels than HCC patients with an AFP level less than or equal to 400 ng/mL (Figure 1(h)). The high expression of HtrA2 in HCC was further confirmed by the GSE121248 (*P* < 0.001), GSE101685 (*P*=0.012), GSE76427 (*P* < 0.001), and GSE76297 (*P* < 0.001) data set from GEO (Figures 1(i)–1(l)).

### 3.2. Upregulation of HtrA2 Is Associated with Poor Prognosis in HCC Patients

**Figure 2:**
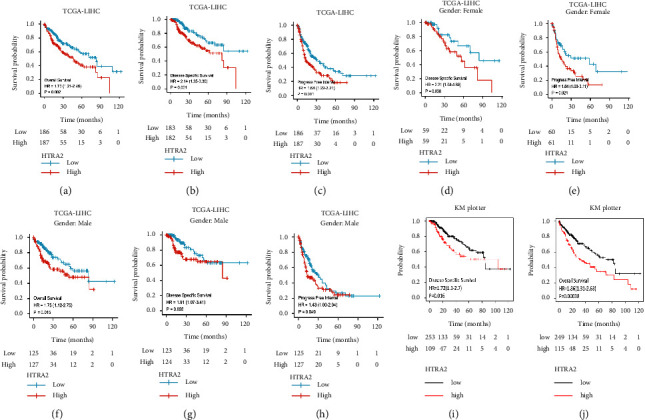
The prognostic value of HtrA2 expression in HCC. (a) Kaplan–Meier OS curves in HtrA2 low and high expression HCC cases from the TCGA database. (b) Kaplan–Meier DSS curves in HtrA2 low and high expression HCC cases from the TCGA database. (c) Kaplan–Meier PFI curves in HtrA2 low and high expression HCC cases from the TCGA database. (d) Kaplan–Meier DSS curves in HtrA2 low and high expression female HCC cases from the TCGA database. (e) Kaplan–Meier PFI curves in HtrA2 low and high expression female HCC cases from the TCGA database. (f) Kaplan–Meier OS curves in HtrA2 low and high expression male HCC cases from the TCGA database. (g) Kaplan–Meier DSS curves in HtrA2 low and high expression male HCC cases from the TCGA database. (h) Kaplan–Meier PFI curves in HtrA2 low and high expression male HCC cases from the TCGA database. (i, j) Survival analyses of HtrA2 by Kaplan–Meier estimator with log-rank test obtained from the Kaplan–Meier plotter web tool. HCC: hepatocellular carcinoma; TCGA: the cancer genome atlas; OS: overall survival; DSS: disease specific survival; PFI: progress free interval.

To assess the clinical relevance of HtrA2 expression in HCC, we explored the survival differences between patients with high and low (grouped according to median) expression of HtrA2 using the Kaplan–Meier method (Figure 2). In the TCGA-HCC cohort, patients with high HtrA2 expression showed shorter overall survival (OS, HR = 1.73, 95% CI: 1.21–2.46, *P*=0.002, Figure 2(a)), disease-specific survival (DSS, HR = 2.14, 95% CI: 1.35–3.39, *P*=0.001, Figure 2(b)), and progression-free interval (PFI, HR = 1.64, 95% CI: 1.22–2.21, *P*=0.001, Figure 2(c)) relative to the patients with low HtrA2 expression. Using the TCGA-HCC data, we performed the survival analysis, separating the HCC patients according to their gender. Female patients with high HtrA2 expression exhibited significantly shorter DSS (HR = 2.21, 95% CI: 1.04–4.69, *P*=0.038, Figure 2(d)) and PFI (HR = 1.84, 95% CI: 1.08–3.11, *P*=0.024, Figure 2(e)) than those female patients with low HtrA2 expression. However, male patients with high HtrA2 expression had shorter OS (HR = 1.75, 95% CI: 1.12–2.75, *P*=0.015, Figure 2(f)), DSS (HR = 1.91, 95% CI: 1.07–3.41, *P*=0.028, Figure 2(g)) and PFI (HR = 1.43, 95% CI: 1.00–2.04, *P*=0.049, Figure 2(h)) than those male patients with low HtrA2 expression. In addition, we checked the Kaplan–Meier Plotter and found that high HtrA2 expression is associated with poor DSS (HR = 1.72, 95% CI: 1.1–2.7, *P*=0.016, Figure 2(i)) and OS (HR = 1.86, 95% CI: 1.31–2.63, *P*=0.00038, Figure 2(j)).

### 3.3. High HtrA2 Expression Is Considered an Independent Risk Factor for HCC

**Figure 3:**
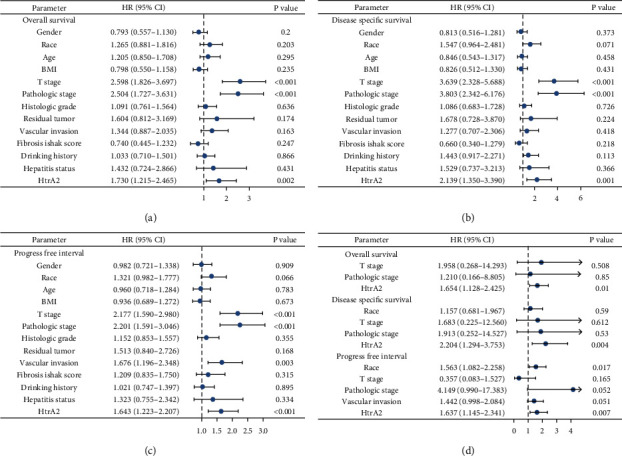
Univariate analysis and multivariate analysis of the correlation of HtrA2 expression and important clinical characteristics with survival among hepatocellular carcinoma patients. (a–c) The exploratory univariate analysis examines the impact of multiple candidate factors on OS, DSS, and PFI. (d) The multivariable Cox regression analysis that included the variables identified through univariable screening for OS, DSS, and PFI. OS: overall survival; DSS: disease specific survival; PFI: progress free interval; HR: hazard ratio; CI: confidence interval.

The prognostic value of HtrA2 expression in HCC patients was further confirmed using the Cox proportional hazards model. Potential prognostic factors in the univariable analysis (Figures 3(a)–3(c)) with *P* < 0.1 were included in the multivariable analysis. The multivariate Cox analyses (Figure 3(d)) showed that only high HtrA2 expression is an important risk factor for OS and DSS. Consistently, in the PFI Cox analysis, HtrA2 and race were acting as potential risk roles in HCC patients.

### 3.4. The Methylation of the HtrA2 Gene Is Associated with HtrA2 mRNA Expression and Predicts Patient Prognosis in HCC

**Figure 4:**
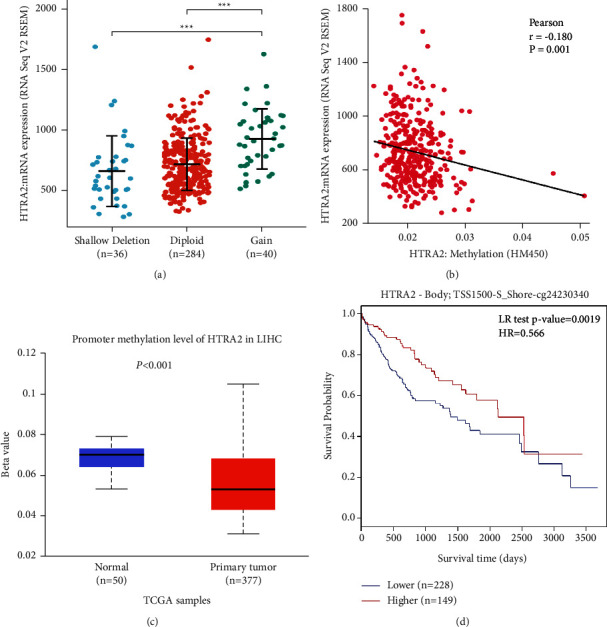
The CNV and methylation of HtrA2 in HCC. (a) Relative mRNA expression of HtrA2 from samples with different CNV statuses (*n* = 360). (b) Relationship between HtrA2 methylation and its expression level (*n* = 373). (c) The promoter methylation of HtrA2 in tumor tissues (*n* = 377) and normal tissues (*n* = 50) from the TCGA-HCC data. (d) The Kaplan–Meier survival of the promoter methylation of HtrA2 in HCC (*n* = 377). CNV: copy number variation; HCC: hepatocellular carcinoma; TCGA: the cancer genome atlas. ^*∗∗∗*^*P* < 0.001.

The data sets on the HtrA2 gene, including mRNA expression data, copy number variation (CNV) data, and methylation data in HCC, were obtained from the cBioPortal for Cancer Genomics. The copy number for each HtrA2 was categorized according to copy number level per gene as shallow deletion, diploid, and gain. Patients with the HtrA2 gene copy number gain had a higher level of HtrA2 expression in HCC, but only 40 of 360 patients (11.1%) had the HtrA2 gene copy number gain (Figure 4(a)). The results suggested that CNV may not be the leading cause of the high expression of HtrA2 in HCC. We further explored the relationship between HtrA2 methylation and gene expression, and these results proved that HtrA2 gene methylation was negatively correlated with gene expression (*r* = −0.18, *P*=0.001) (Figure 4(b)). Based on the UALCAN database, we found that promoter regions of HtrA2 were shown to be significantly less methylated in tumor tissues of HCC than in the normal tissues adjacent to cancer (*P* < 0.001, Figure 4(c)). In addition, methylation of HtrA2 was strongly associated with survival in HCC patients, and hypomethylation was associated with a negative prognosis (HR = 0.566, *P*=0.0019) (Figure 4(d)).

### 3.5. HtrA2 Coexpression Networks in HCC

**Figure 5:**
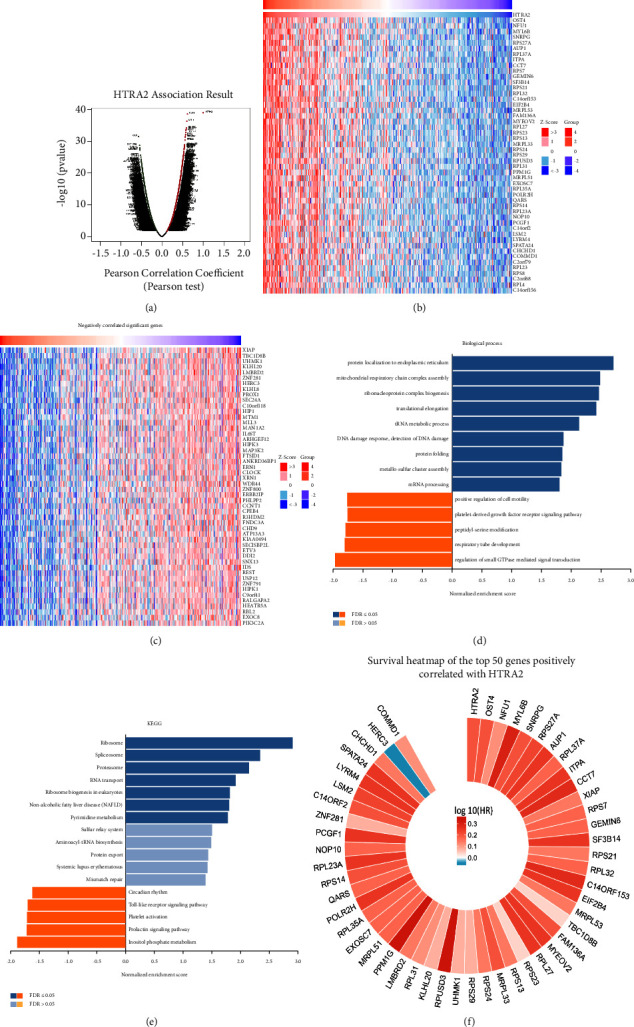
HtrA2 coexpression genes in HCC (LinkedOmics). (a) The global HtrA2 highly correlated genes identified by the Pearson test in HCC. (b) The heatmap showing the top 50 genes positively correlated with HtrA2 in HCC. (c) The heatmap shows the top 50 genes negatively correlated with HtrA2 in HCC. (d, e) Significantly enriched GO: biological process annotations and KEGG pathways of HtrA2 in HCC. (f) The survival heatmap of the top 50 genes positively correlated with HtrA2 in HCC. The survival heatmap shows the hazard ratios in the logarithmic scale (log10) for different genes. FDR: false discovery rate; KEGG: kyoto encyclopedia of genes and genomes; GO: gene ontology; HCC: hepatocellular carcinoma.

To obtain a deep understanding of the biological meaning of HtrA2 in HCC, the “LinkFinder” module in LinkedOmics was used to detect the coexpression pattern of HtrA2. The results identified 4843 genes (red dots) positively correlated with HtrA2 and 6070 genes (green dots) negatively correlated (*P* value <0.05) (Figure 5(a)). Heat maps showed the top 50 genes significantly positively and negatively correlated with HtrA2, respectively (Figures 5(b) and 5(c)). Gene ontology enrichment was determined using the GSEA. The results showed that HtrA2 coexpressed genes involved mainly in protein localization to endoplasmic reticulum, mitochondrial respiratory chain complex assembly, ribonucleoprotein complex biogenesis, translational elongation, tRNA metabolic process, DNA damage response, protein folding, metallo-sulfur cluster assembly, and mRNA processing. However, positive regulation of cell motility, platelet-derived growth factor receptor signaling pathway, peptidyl-serine modification, respiratory tube development, and small GTPase-mediated signal transduction regulation were inhibited (Figure 5(d)). KEGG enrichment analysis showed that HtrA2 coexpressed genes were primarily enriched in the ribosome, spliceosome, proteasome, RNA transport, ribosome biogenesis in eukaryotes, and nonalcoholic fatty liver disease (NAFLD) (Figure 5(e)). Interestingly, the top 50 genes positively coexpressed with HtrA2, except for HERC3, were all independent prognostic indicators in HCC, and each of these genes had a high hazard ratio (HR, *P* value <0.05) (Figure 5(f)). In addition, the HtrA2 coexpressed genes in HCC were analyzed using the GSEA method. Results demonstrated that the top 5 microRNAs were miR-186, miR-527, miR-26A, miR-519E, and miR-30A-5P (Table 1). Transcription factor enrichment analysis showed that the HtrA2 coexpressed genes were mainly related to V$IPF1_Q4, V$HFH4_01, PAX4_02, V$E4BP4_01, and V$ETF_Q6 (Table 1).

### 3.6. The Correlation between HtrA2 Expression and the Levels of Infiltrating Immune Cells

**Figure 6:**
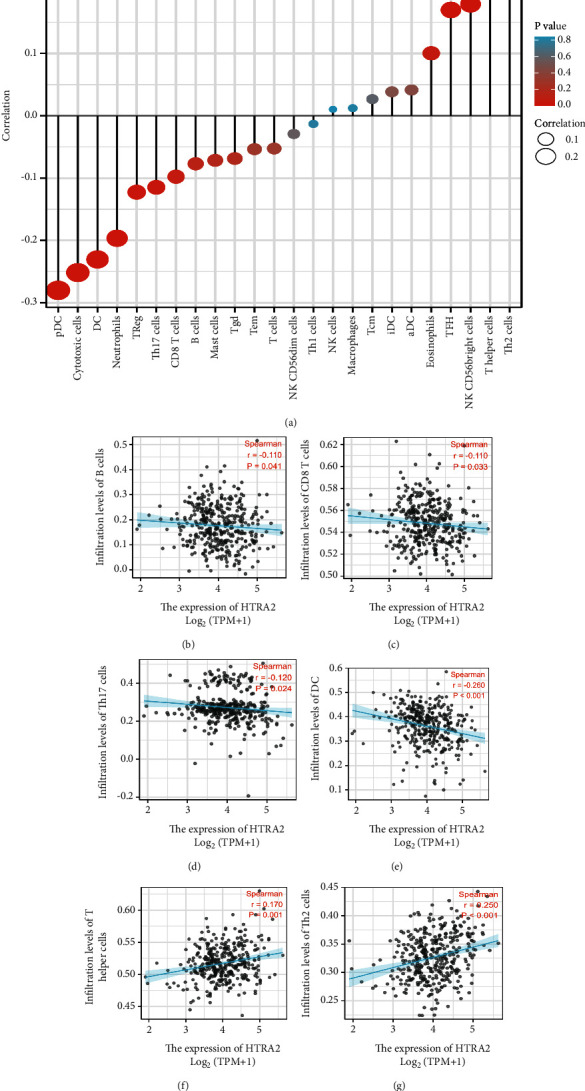
Correlation analyses between HtrA2 expression and immune infiltration level in HCC. (a) The correlation between the infiltration of immune cells and the expression of HtrA2. (b–e) HtrA2 expression significantly negatively correlates with infiltrating levels of B cells, CD8 T cells, Th17 cells, and dendritic cells. (f, g) HtrA2 expression significantly positively correlates with infiltrating levels of T helper cells and Th2 cells. HCC: hepatocellular carcinoma; DC: dendritic cells.

**Figure 7:**
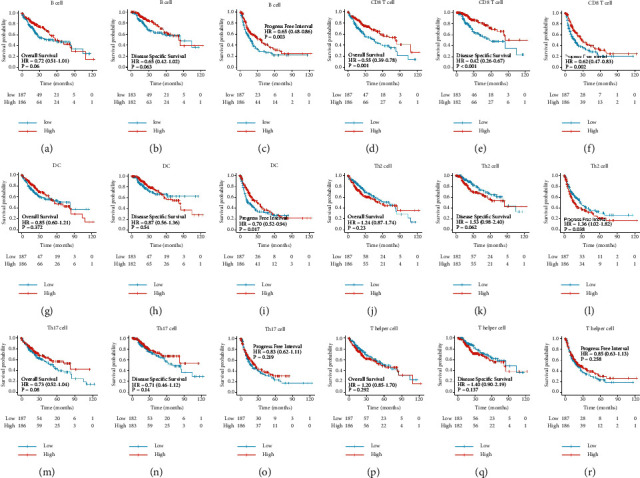
Survival analyses of filtered immune cells in HCC. The difference in OS between different infiltration levels of B cells (A), CD8 T cells (D), DC(G), Th2 cells (J), Th17 cells (M), and T helper cells (P), separately. The difference in DSS between different infiltration levels of B cells (B), CD8 T cells (E), DC (H), Th2 cells (K), Th17 cells (N), and T helper cells (Q), separately. The difference in PFI between different infiltration levels of B cells (C), CD8 T cells (F), DC (I), Th2 cells (L), Th17 cells (O), and T helper cells (R), separately. HCC: hepatocellular carcinoma; DC: dendritic cells; OS: overall survival; DSS: disease specific survival; PFI: progress free interval.

Because the immune system might play a key role in developing HCC, we further analyzed the relationship between HtrA2 mRNA expression and the levels of infiltrating immune cells. The correlation between HtrA2 mRNA expression and the infiltration of immune cells is shown in Figure 6(a). The results showed that the HtrA2 mRNA expression was significantly and negatively correlated with the infiltration of B cells (Figure 6(b)), CD8 T cells (Figure 6(c)), Th17 cells (Figure 6(d)), and dendritic cells (DC) (Figure 6(e)). In addition, HtrA2 mRNA expression also positively correlated with T helper cells (Figure 6(f)) and Th2 cells (Figure 6(g)). In addition, we evaluated the prognostic impact of each of the six types of immune cells on OS, DSS, and PFI (Figures 7(a)–7(r)), finding that the infiltration levels of B cells, CD8 T cells, DC, and Th2 cells can predict the outcome of HCC. Among them, CD8 T cell infiltration level was considered the most specific marker for prognosis prediction. Therefore, CD8 T cell infiltration may be the key pathological mechanism by which HtrA2 influences the outcome of HCC.

### 3.7. Validation of HtrA2 Expression in HCC

**Figure 8:**
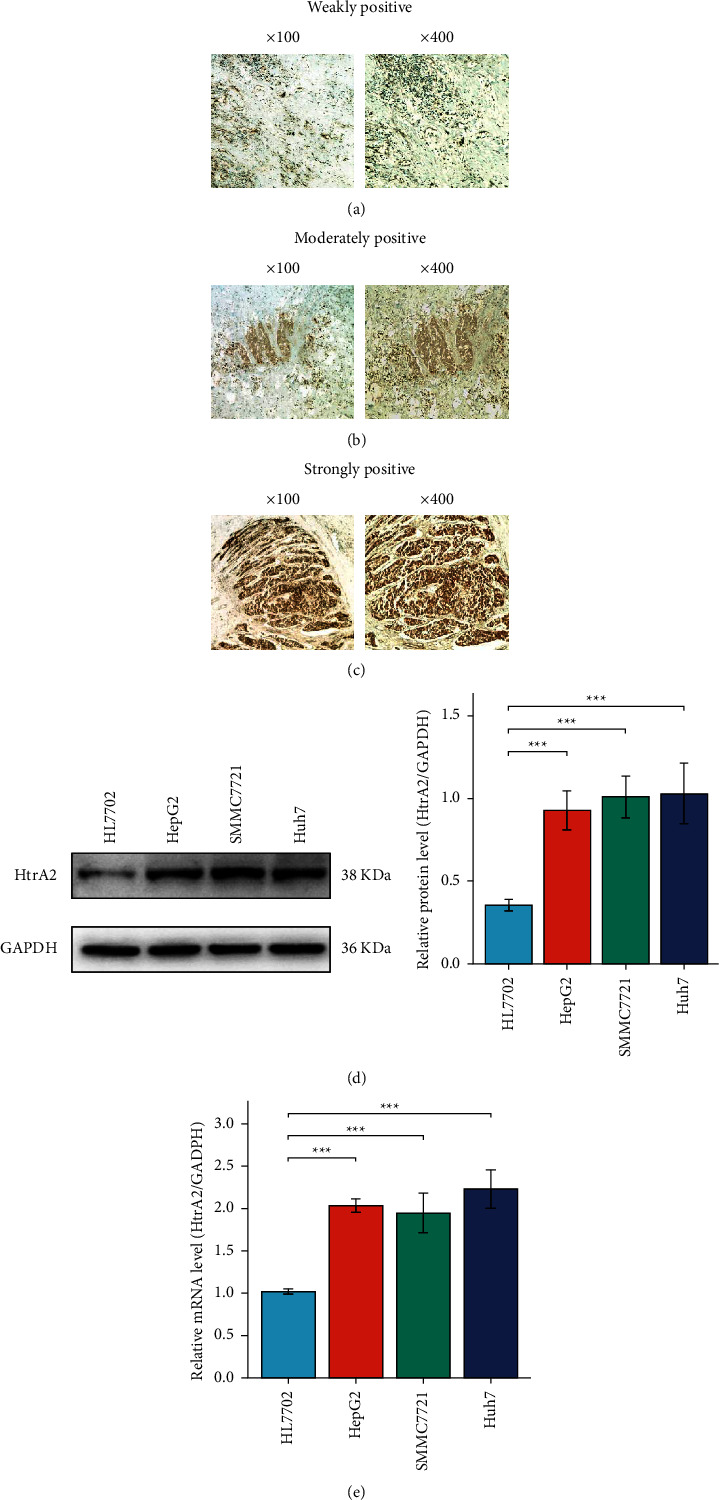
HtrA2 mRNA and protein expression in cell lines and HCC tissues. (a–c) Representative immunohistochemistry staining patterns of HtrA2 in HCC tissues. (d) HtrA2 protein expression in a normal human liver cell line and three HCC cell lines. (e) HtrA2 mRNA expression in a normal human liver cell line and three HCC cell lines. HCC: hepatocellular carcinoma. ^*∗∗∗*^*P* < 0.001.

According to the results of IHC, HtrA2 protein expression in HCC was divided into weakly (Figure 8(a)), moderately (Figure 8(b)), and strongly positive (Figure 8(c)) as shown in Figure 8. The results also showed that HtrA2 mRNA and protein expression in HCC cell lines (HepG2, SMMC7721, and Huh7) was significantly higher than that of HL7702 (all *P* < 0.001, Figures 8(d) and 8(e)).

## 4. Discussion

In the present study, HtrA2 was found to have high expression in HCC tumor tissue, and its high expression was associated with hypomethylation. In addition, HtrA2 mRNA expression and hypomethylation of HtrA2 were both associated with a poor prognosis in HCC. Univariate and multivariate analyses confirmed that the high expression of HtrA2 might be an independent prognostic factor in patients with HCC. We next explored the potential coexpression and regulatory networks of HtrA2. Furthermore, we examined the relationships between HtrA2 and immune cell infiltration, finding that overexpression of HtrA2 in HCC was associated with the infiltration of various immune cells. At last, we experimentally confirmed the expression level of HtrA2 in HCC. This study sheds new light on the understanding of the role of HtrA2 in HCC development and provides directions for further research on the management of HCC.

According to the results of previous studies, higher expression of HtrA2 had been found in malignant thyroid tumors, gastric cancer, and prostate cancer, and it was predictive of poor patient outcomes [27–30]. However, the expression of HtrA2 was found to be very low, which predicted poor outcomes in non-small-cell lung cancer and ovarian cancer [31, 32]. These findings reflect the differential roles of HtrA2 in different tumor tissues and stages of tumor progression.

Based on the TCGA database, GEO data sets, and our experimental data, this study found that the expression of HtrA2 was significantly higher in HCC than in normal liver tissues. We also found that HtrA2 was significantly overexpressed in multiple cancers in the TCGA data, which is consistent with previous studies. Therefore, HtrA2 has the potential to be a diagnostic marker for multiple tumor types, including HCC. According to our results, the diagnostic performance of HtrA2 in HCC detection was promising, with an AUC value of 0.917. Furthermore, the study demonstrated that a high HtrA2 was correlated with a more advanced pathologic stage, higher AFP level, and histologic grade in HCC. These results indicated that HtrA2 might be related to the malignant degree and growth of HCC. Survival analysis results also indicated that higher expression of HtrA2 portended a poor prognosis for patients with HCC. However, the predictive value of HtrA2 seemed to depend on the patient's sex. HtrA2 had a better predictive value for OS in male patients than in female patients. In addition, Cox regression analysis further proved that HtrA2 was an independent predictor of adverse clinical outcomes in HCC patients. The present study also explored the mechanism of HtrA2 mRNA overexpression in HCC, and the results of our study showed that DNA hypomethylation was a possible mechanism contributing to HtrA2 mRNA overexpression in HCC.

Interestingly, HtrA2 methylation states were also related to the prognosis of HCC, as HtrA2 methylation levels were negatively correlated with overall survival times in HCC patients. These findings were consistent with our previous conclusion that high HtrA2 expression was associated with a poor prognosis in HCC patients. Although many mechanisms are likely to contribute to the overexpression of HtrA2 in HCC, hypomethylation may be one of the major regulatory mechanisms.

Previous studies support the conclusion that HtrA2 not only acts as an apoptotic-inducing protein but also contributes to removing denatured proteins from the mitochondria following exposure to heat shock or other stresses [33]. Functional enrichment analysis of coexpressed genes of HtrA2 found that HtrA2 might participate in multiple biological processes (such as protein localization to the endoplasmic reticulum, DNA damage response, and protein folding) and important protein synthesis pathways (including ribosome and ribosome biogenesis). These results are generally consistent with previous publications. As with HtrA2, many genes positively coexpressed with HtrA2 have a strong and independent prognostic role for HCC patients. This is further evidence that HtrA2 plays an important role in HCC prognosis.

We further explored the regulators responsible for HtrA2 dysregulation and found that HtrA2 was related to the network of microRNAs, such as miR-186, miR-527, miR-26A, miR-519E, and miR-30A-5P. miR-186 shows low expression in liver cancer stem cells, and its overexpression inhibits liver cancer stem cell self-renewal and tumorigenesis [34]. Upregulation of miR-186 may inhibit the nuclear *β*-catenin accumulation and the activation of Wnt/*β*-catenin signaling in HCC cells, reducing tumor cell proliferation and metastasis [35]. Furthermore, the highly upregulated long noncoding RNA can block the inhibitory effects of miR-186 on high mobility group A2, a validated oncogene in HCC [36]. Nonetheless, the exact pathogenetic mechanisms by which decreased miR-186 expression promotes hepatocarcinogenesis remain unclear. Several studies have confirmed the importance of miR-527-mediated gene regulation in HCC development. For example, miR-527 inhibits HCC tumorigenesis by regulating its targets, including UBE2A, FBXW7, and glypican-3 [37]. It has been well established in biomedical research that miR-26A suppresses tumor progression in multiple cancers, such as colorectal cancer, gastric cancer, and HCC [38–40]. Yang et al. show that miR-26A regulates Janus kinase 1 to inhibit HCC cell proliferation, invasion, and metastasis [41]. Mahati et al. found that nanosystem-mediated miR-26a delivery can inhibit the proliferation and migration of glypican-3-positive HCC cells in vitro and show an improved therapeutic effect in liver cancer xenografted mouse models [42]. Likewise, miR-30A-5P also has an important role in the development of HCC. Zhang et al. discovered that miR-30a-5p/CLCF1 could regulate sorafenib resistance in HCC, and treatment with cholesterol-modified agomiR-30a-5p significantly reduced tumor growth in mice harboring sorafenib-resistant HCC tumors [43]. Moreover, miR-30a-5p upregulation can block the enhanced migration and invasion of HCC cells induced by lncRNA loc339803 overexpression [44]. Pan et al. found that vimentin is a target of miR-30a-5p, and the miR-30a-5p-Vimentin axis is a potential molecular biomarker and therapeutic target in HCC [45]. Previous studies showed that miR-519E regulates tumor cell apoptosis, proliferation, and migration [46, 47]. Since HtrA2 can regulate tumor cell apoptosis, there is a possible connection between miR-519E and HtrA2. The above evidence may provide clues for further exploration of the relationship between HtrA2 and the network of microRNAs in HCC. This study found that HtrA2 dysregulation was also associated with multiple transcription factors, including IPF1, HFH4, PAX4, E4BP4, and ETF. All the major transcription factors regulate the cell apoptosis and proliferation of different types of human cells [48–52]. Our results indicate that HtrA2 may regulate the cell apoptosis and proliferation of HCC through these factors.

We found that the expression of HtrA2 was significantly associated with the immune infiltrate, and CD8 T cell infiltration might be one of the critical factors of HtrA2 with prognostic value in HCC. The results were consistent with those from studies in other tumor types. Hu et al. reported that HtrA2 regulates CCR2-mediated breast cancer cell growth and cellular invasion in a CCL2/CCR2 context-dependent manner [53]. While the importance of CCL2/CCR2 signaling in macrophages during cancer progression is well documented, HtrA2 may regulate macrophage recruitment. In addition, previous studies found a strong relationship between the expression of HtrA2 and the expression of immune inhibitors (CD274, IDO1, TIGIT, etc.), immunostimulators (CD80, CD86, ICOS, etc.), and chemokines (CCL2, CCL7, CXCL9, etc.). Not only so, HtrA2 tends to express mainly at CD8 T, DC, plasma, fibroblasts, mast, and malignant cell clusters, possibly indicating that HtrA2 also functions in immune cells or stromal cells other than cancer cells [24]. However, studies on the correlation between HtrA2 and tumor immune infiltration are currently lacking. More progress will be made in this research field for the foreseeable future.

Nevertheless, the current study also has some shortcomings, which are described as follows. (1) Our study is limited by its retrospective data, and the findings need to be confirmed by prospective studies. (2) Due to incomplete data, we were unable to assess the association of HtrA2 expression with the clinical staging of liver cancer, including the Barcelona Clinic Liver Cancer (BCLC) stage and China Liver Cancer staging (CNLC). (3) The lack of appropriate in vivo and in vitro experimental data hampered the understanding of the role of HtrA2 in the HCC. This is, of course, a problem faced by other similar studies [54, 55]. We have cultivated cell lines ready for further wet-lab experimentation to validate our results and investigate additional critical signaling pathways associated with HtrA2 in the HCC.

In summary, HtrA2 mRNA expression was overexpressed in HCC, while methylation of HtrA2 was decreased in HCC. Poor prognosis in HCC is associated with high HtrA2 mRNA expression and low HtrA2 methylation. Our results also showed that HtrA2 might play a key role in modulating the proliferation and apoptosis of HCC cells, possibly by interacting with microRNAs and multiple transcription factors. Furthermore, the infiltration of most immune cells is significantly associated with HtrA2 expression, suggesting a possible involvement of HtrA2 in regulating the tumor immune response. This study demonstrated that HtrA2 has the potential to be a prognostic and diagnostic HCC biomarker, highlighting the potential therapeutic value of HtrA2 as an anticancer drug target.

## Figures and Tables

**Table 1:** The miRNAs and transcription factors-target networks of HtrA2 in HCC.

Enriched categories	Gene set	Leading edge number	NES	FDR
miRNA target	ATTCTTT, miR-186	99	−2.1062	0
	CTTTGCA, miR-527	113	−2.1136	0
	TACTTGA, miR-26A, and miR-26B	119	−2.1139	0
	GGCACTT, miR-519E	55	−2.1145	0
	TGTTTAC, miR-30A-5P, miR-30C, miR-30D, miR-30B, and miR-30E-5P	203	−2.1173	0

Transcription factor	V$IPF1_Q4	83	−1.8582	0.00066
	V$HFH4_01	83	−1.8681	0.00083
	V$PAX4_02	76	−1.8692	0.00111
	V$E4BP4_01	84	−1.8904	0.00166
	V$ETF_Q6	36	−1.8111	0.00194

HCC: hepatocellular carcinoma; NES: normalized enrichment score; FDR: false discovery rate; HtrA2: high-temperature requirement protein A2; miRNAs: microRNAs.

## Data Availability

Raw data are available from the corresponding author upon request.

## Supplementary Materials

Supplementary Materials Supplementary table 1: abbreviation list. An abbreviation list was provided for ease of reading.
