# Supplementary material for: HtrA2 Independently Predicts Poor Prognosis and Correlates with Immune Cell Infiltration in Hepatocellular Carcinoma
Source: J Oncol. 2023 Jan 17;2023:4067418. doi: 10.1155/2023/4067418 (PMC9873461; doi:10.1155/2023/4067418)
Supplement: Supplementary Materials — Supplementary table 1: abbreviation list. An abbreviation list was provided for ease of reading. [file 4067418.f1.docx]

| **Abbreviations** | **Full names of abbreviations** |
| --- | --- |
| HtrA2 | High-temperature requirement protein A2 |
| HCC | Hepatocellular carcinoma |
| TCGA | The cancer genome atlas |
| GEO | Gene expression omnibus |
| ROC curve | Receiver operating characteristic curve |
| CNV | Copy number variation |
| KEGG | Kyoto Encyclopedia of Genes and Genomes |
| GSEA | Gene set enrichment analysis |
| ssGSEA | Single-sample Gene Set Enrichment Analysis |
| BRCA | Breast carcinoma |
| BLCA | Urothelial bladder carcinoma |
| CHOL | Cholangiocarcinoma |
| COAD | Colon adenocarcinoma |
| ESCA | Esophageal carcinoma |
| GBM | Glioblastoma multiforme |
| HNSC | Head-neck squamous cell carcinoma |
| KIRC | Kidney renal clear cell carcinoma |
| KIRP | Kidney renal papillary cell carcinoma |
| LIHC | Hepatocellular carcinoma |
| LUAD | Lung adenocarcinoma |
| LUSC | Lung squamous cell carcinoma |
| PCPG | Pheochromocytoma and paraganglioma |
| PRAD | Prostate adenocarcinoma |
| READ | Rectum adenocarcinoma |
| STAD | Stomach adenocarcinoma |
| THCA | Thyroid carcinoma |
| UCEC | Uterine corpus endometrial carcinoma |
| OS | Overall survival |
| DSS | Disease specific survival |
| PFI | Progression free interval |
| NAFLD | Non-alcoholic fatty liver disease |
| HERC3 | HECT and RLD domain containing E3 ubiquitin-protein ligase 3 |
| DC | Dendritic cells |
| Th cells | T helper cells |
| NES | Normalized enrichment score |
| FDR | False discovery rate |
| miRNAs | Micrornas |
| UBE2A | Ubiquitin conjugating enzyme e2a |
| FBXW7 | F-Box and WD Repeat Domain Containing 7 |
| CLCF-1 | Cardiotrophin-like cytokine factor 1 |
| IPF1 | Insulin promoter factor 1 |
| HFH4 | HNF-3/fork head homolog-4 |
| PAX4 | Paired box 4 |
| E4BP4 | E4 promoter-binding protein |
| ETF | Electron-transfer flavoprotein |
| CCR2 | C-C chemokine receptor type 2 |
| CCL2 | Chemokine (C-C motif) ligand 2 |
| CXCL9 | Chemokine (C-X-C motif) ligand 9 |
| BCLC stage | Barcelona Clinic Liver Cancer stage |
| CNLC staging | China Liver Cancer staging |

**Supplementary table 1 Abbreviation list**
